# Eyelid retraction during smiling in a patient with monocular congenital ptosis: a case report

**DOI:** 10.1186/s12886-024-03485-8

**Published:** 2024-05-31

**Authors:** Yiyang Zhao, Jing Fu, Jie Hao

**Affiliations:** 1 Beijing Tongren Eye Center, Beijing Tongren Hospital, Capital Medical University, Beijing, 100730 China; 2 Department of Strabismus and Pediatric Ophthalmology, Beijing Tongren Eye Center, Beijing Tongren Hospital, Capital Medical University, Beijing, 100730 China

**Keywords:** Congenital ptosis, Impaired innervation, Synkinesis, Levator palpebrae superioris, Lid retraction

## Abstract

**Background:**

Blepharoptosis is a common symptom in ophthalmology clinic, but eyelid retraction when smiling in a ptosis eye is a rare manifestation. Here we report a novel manifestation that eyelid retraction during smiling in a patient with monocular congenital ptosis.

**Case description:**

A 10-year-old girl with isolated and mild unilateral congenital ptosis showed eyelid retraction in ptotsis eye when smiling together with a lid lag on downgaze. She didn’t have any systematic and ocular diseases other than myopia and astigmatism.Eyelid retraction during smiling is 5 mm, resulting in a significant difference in the height of bilateral palpebral fissures.As for ptosis, is mild.The margin to reflex distance 1 is 1.0 mm on the right eye(ptosis eye) and 3.0 mm on the left eye. A lid lag of 1.0 mm on downward gaze was noted on the right, she could close her eyes fully while sleeping.The ice pack test, laboratory test for thyroid function, whole-exome sequencing (WES) and magnetic resonance imaging(MRI) of the orbital and ocular motor nerves showed normal results.Her symptoms alleviated after 6 months, with the retraction of the right upper eyelid when smiling was approximately 3 mm, thus the difference in the palpebral fissure height when smiling was smaller than that at the initial presentation.

**Conclusion:**

Blepharoptosis may accompanied with abnormal innervation like eyelid retraction, this phenomenon can be alleviated with age.The results of the levator muscle function test should be carefully examined to determine whether it is ptosis in an impaired innervation eyelid.

**Supplementary Information:**

The online version contains supplementary material available at 10.1186/s12886-024-03485-8.

## Background

Blepharoptosis, often abbreviated as ptosis, is a condition in which an upper eyelid is positioned lower than the normal position, resulting in the narrowing of the vertical dimension of the palpebral fissure [1]. Unilateral ptosis is caused by degenerative, traumatic, myogenic, neurogenic, and mechanical pathological issues [2]. Recent studies suggest that unilateral ptosis has a neurogenic origin and is probably secondary to impaired innervation of the levator palpebrae muscle [3]. The condition presents as a stiff ptosis eyelid with poor contraction function and incomplete relaxation, identical to lid lag. Furthermore, similar to Marcus Gunn jaw-winking syndrome, synkinesis with ptosis is a rare condition with an association between abnormal innervation of the levator palpebrae superioris muscle and the impaired movement of the mouth or jaws; this condition is similar to that reported in this case [4]. Although the surgical process for ptosis has advanced and further improved, the surgical methods for monocular congenital ptosis with synkinesis or impaired innervation vary and remain controversial; this is mostly because of lagophthalmos after surgery. Congenital ptosis with impaired innervation can be treated in expectant treatment in patients without visual abnormalities such as amblyopia; this is because developmental issues such as impaired innervation may improve with age. Here, we present a novel manifestation of monocular congenital ptosis with impaired innervation in a 10-year-old girl with isolated and mild unilateral congenital ptosis; the patient showed eyelid retraction when smiling along with a lid lag on downgaze. After 6 months, the abnormal symptoms were alleviated.

At the time of writing, there have not been any reports of this presentation in the literature including PUBMED and Cochrane databases. This report was carried out in accordance with the ethical principles outlined in the Declaration of Helsinki 2013 and written consent was obtained from the patient.

**Fig. 1:**
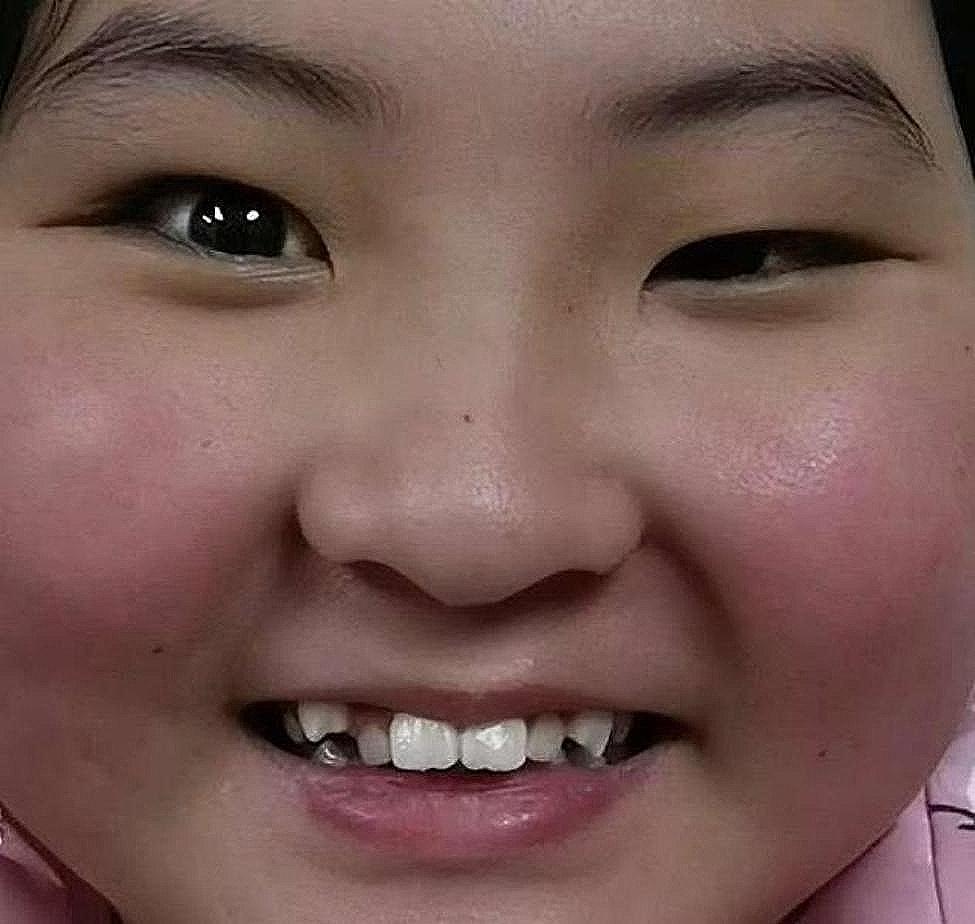
Facial photograph shows right upper eyelid retraction during smiling is 5 mm, resulting in a significant difference in the height of bilateral palpebral fissures

**Fig. 2:**
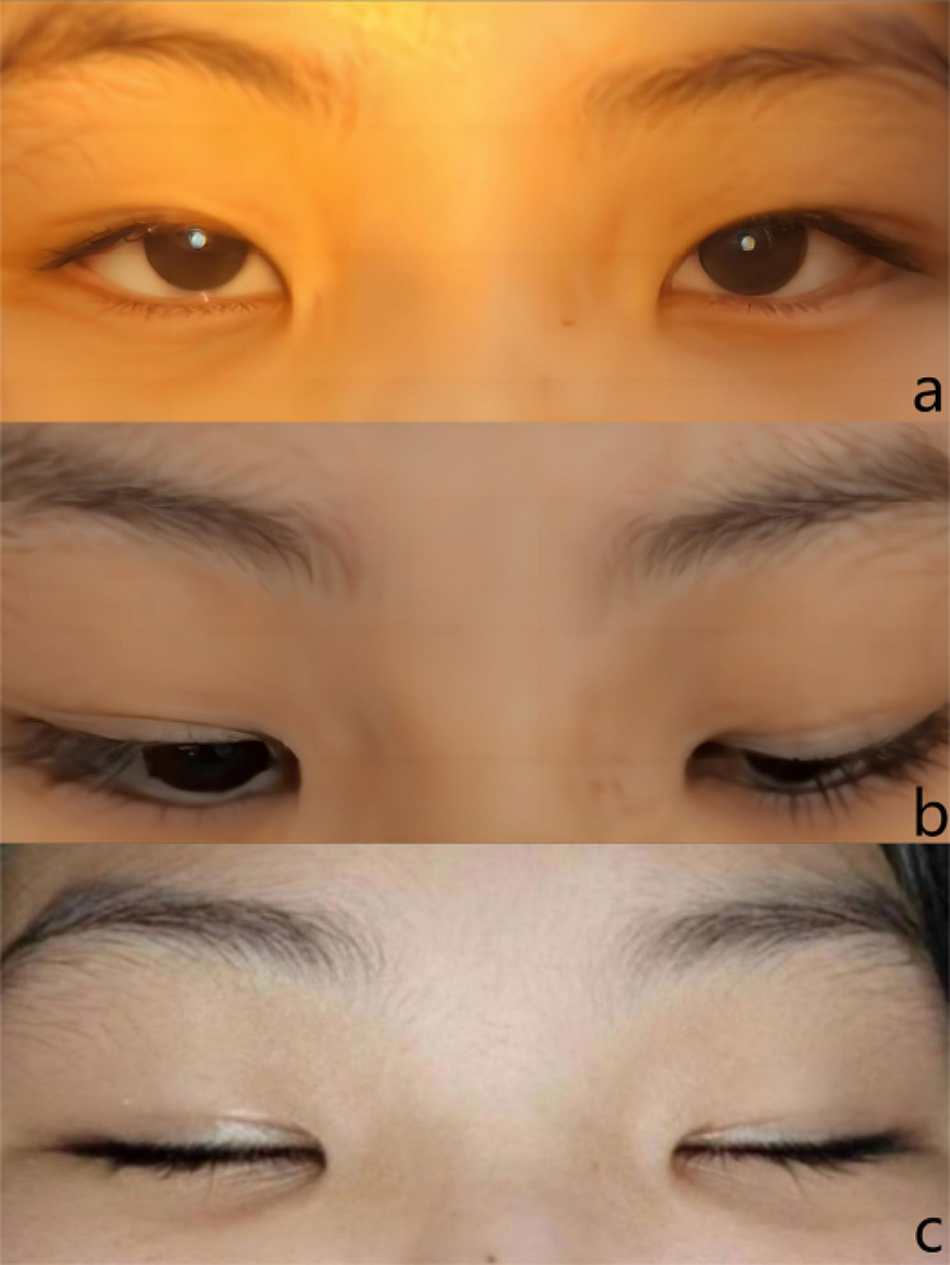
The margin to reflex distance 1 is 1.0 mm on the right eye and 3.0 mm on the left eye (Panel a). A lid lag of 1.0 mm on downward gaze was noted on the right (Panel b). The girl could close her eyes fully while sleeping (Panel c)

## Case description

A 10-year-old girl visited our clinic. Her parents complained that their surrogate daughter appeared abnormal, with a marked difference in the height of the bilateral palpebral fissure when smiling after she was born (Fig. 1). The left upper eyelid seemed to have ptosis. The patient did not complain about pain, diplopia, muscle weakness, etc. She also denied a history of birth injury and hereditary ocular diseases. She could close her eyes fully while sleeping and blinking.

Her parents recalled that the patient was born with her left eye open and right eye closed and that her father used his hand to open the upper and lower eyelids of the right eye. The patient was delivered at 36^+ 20^ weeks, and her birth weight was 3.5 kg. The girl had an older brother with no ocular or systemic diseases other than low myopia.

During the initial examination, the cycloplegic refraction was–0.50DS – 0.75DC × 175-OD and plano lens-OS. The bilateral corrected visual acuity was 20/20, with normal intraocular pressure, extraocular movement, alignment, and pupil profile. Anterior and posterior segment examination under slit-lamp revealed no abnormal findings. Her visual function was normal, with 60arc sec in the random dot stereo test. Notable ptosis was observed in the right upper eyelid (Fig. 2a), with the margin to reflex distance 1 of 1.0 mm on the right and 3.0 mm on the left. The retraction of the right upper eyelid when smiling was approximately 5 mm; thus, it indicates medium retraction. The right and left levator palpebrae superioris muscle measured 7.0 and 12.0 mm, respectively; a 1.0 mm lid lag on downward gaze was noted on the right eye (Fig. 2b). Her right upper eyelid crease was normally formed and symmetrical with that of her left upper eyelid. Her fundi were normal, and her eyelid movements were full, but poor right levator function was noted in upgaze and insufficient upper eyelid descent in downgaze. The ice pack test and laboratory test for thyroid function showed normal results. Here, daily photographs show that she could close her eyes fully while sleeping (Fig. 2c). The patient was then asked to wear glasses and undergo further examinations.

After 1 month, she underwent complete magnetic resonance imaging (MRI) of the orbital and ocular motor nerves. The findings showed normal morphology of the nerves. Facial electromyography showed normal motor nerve conduction; however, the conduction latency of the levator superior muscle in the right eye was longer than that in the left eye. Negative results were noted for the test of repetitive nerve stimulation(RNS) and blink reflex. She also underwent a neurological examination, and the results revealed no abnormalities in the areas innervated by the facial nerve and trigeminal nerve; however, the levator palpebrae superior muscle innervated by the oculomotor nerve was probably considered to have developmental problems.

**Fig. 3:**
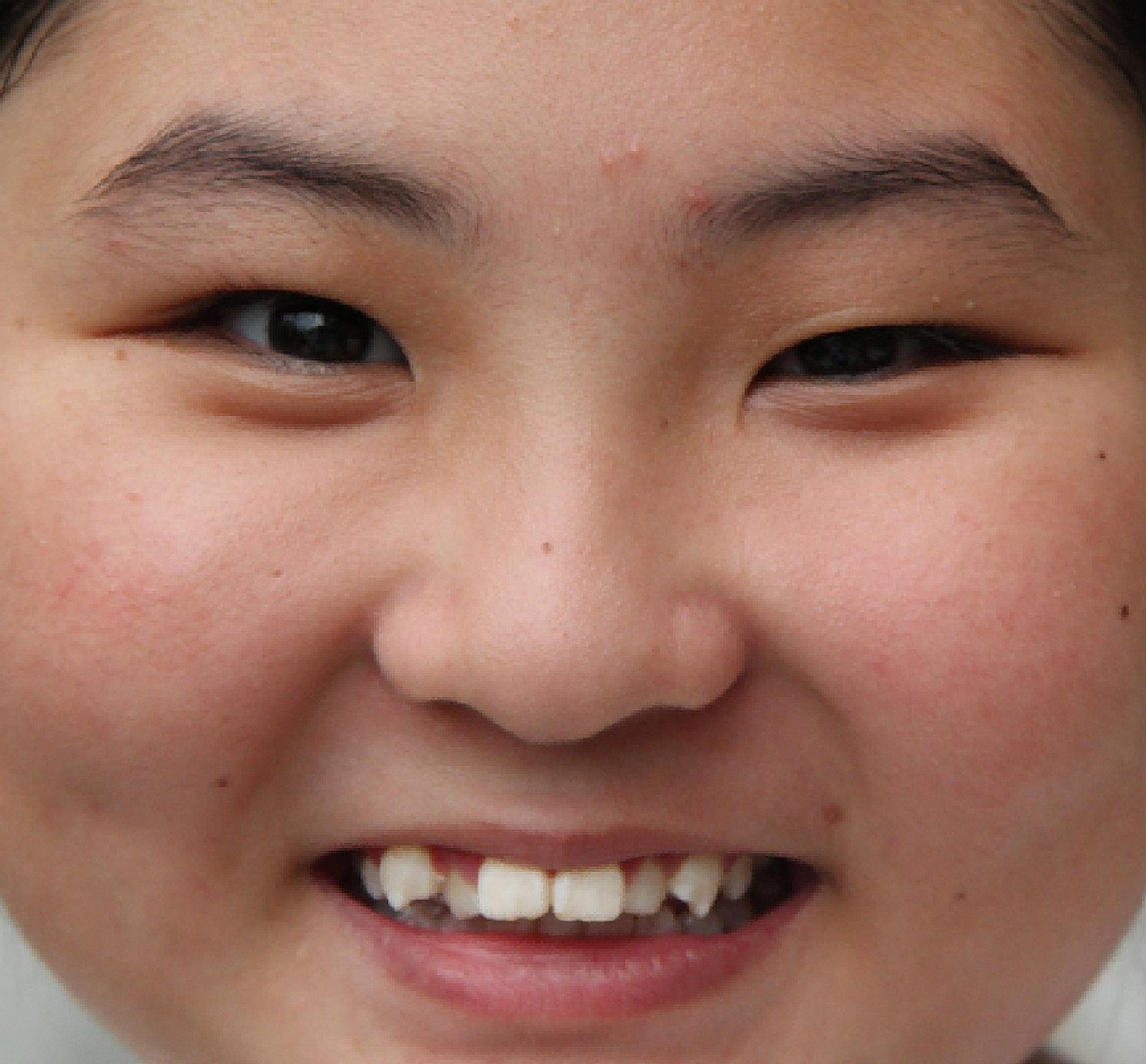
After 6 months, the retraction of the right upper eyelid when smiling was approximately 3 mm. Thus, the difference in the palpebral fissure height when smiling was smaller than that at the initial presentation

At 6-month follow-up, her right upper eyelid retraction during smiling seemed to be milder. The retraction of the right upper eyelid during smiling was approximately 3 mm, not 5 mm (measured 6 months ago). Moreover, a smaller difference was noted in palpebral fissure height than that at the initial presentation. The patient and her parents reported an improvement in appearance (Fig. 3). Ptosis did not seem to change during this period; the margin to reflex distance 1 was approximately 1.3 mm on the right eye and 3.0 mm on the left eye. Lid lag was 1 mm and showed no improvement. The cycloplegic refraction reading was − 1.00DS − 1.00DC × 170-OD and + 0.50DC × 90-OS, the bilateral corrected visual acuity was 20/20. We asked the patient to use new glasses and wear them continuously. Her parents believed that their daughter’s appearance had improved and chose to continue the follow-up rather than surgery.Our patient and her parents agreed that her photos and information can be used for publication purposes.

## Discussion

Recent findings suggest that congenital ptosis is probably secondary to impaired innervation of the levator palpebrae muscle [4]. A case report on an infant with congenital blepharoptosis retraction of the right eyelid supported the phenomenon of ptosis with impaired innervation. In our patient, the right upper eyelid ptosis was accompanied by retraction during smiling. Her ptosis was mild and did not cover the center of sight. Therefore, she only had low myopia with astigmatism on her ptosis eye. Compared to the above-mentioned case report, our patient showed mild ptosis, except that our patient could close her eyes fully while daily sleeping and blinking.

Upper eyelid retraction when smiling is a rare manifestation of congenital isolated ptosis. In the present case, blepharoptosis was extremely insidious, and the contraction of the upper eyelid when smiling covered up right eye ptosis. Similar to Marcus Gunn syndrome, impaired innervation is a rare condition in the field of ophthalmology [5]. We assumed that our patient had congenital synkinesis, whose pathogenesis is similar to that of Marcus Gunn syndrome: the only difference is that there is an abnormal association between the levator palpebrae superioris and the movement of mouth or jaws in Marcus Gunn syndrome, while an association was observed between the ptotic eyelid and the nerve impulse of “smile” in our patient.

The mechanisms of Marcus Gunn jaw-winking syndrome (MGJW) remains unclear. However, there are some hypotheses, electromyographic and histological studies [6, 7] suggest that the process responsible for the Marcus Gunn phenomenon consists of neurogenic atrophy with aberrant reinnervation which occurs in utero. It would also indicate that the initiating pathological process was probably located within the central nervous system, rather than being a peripheral.It has been reported in Xenopus toad that temporal muscle afferent Vme(mesencephalic trigeminal nucleus neurons)neurons project to the III/IV motoneurons directly and this neuronal circuit may be a “primitive” trigemino-oculomotor synkinetic reflex pathway which also exists in healthy human, it is most likely shut off in normal environment, but “released” under certain disease conditions such as MGJW [8]. In recent years, a new hypothesis [9, 10] occurs that the discharge of masseter afferent Vme neurons would probably co-fire the neighbor eyelid afferent Vme neurons, and consequently excite the levator palpebrae and superior rectus motoneuron through the III projecting Vme neurons to motivate a jaw-eyelid synkinesis—the hallmark facial behavior of the MGJW.

Some authors believe that synkinetic retraction fades with age in patients with Marcus Gunn syndrome [11, 12]. Our patient showed eyelid retraction in ptosis eye while smiling when she was born, and her synkinesis symptom decreased during the 6-month follow up.The correct reflexes may have been established through thousands of practices during individual development.Initially, we assumed that it would be the result of “primitive reflex”connected the facial nucleus(VII) and oculomotor nucleus(III) as the synkinesis circuit in MGJW is between V and III, in Marin Amat syndrome is between V and VII [13, 14].Could there also be an aberrant innervation between III and VII The second hypothesis is that the nerve impulse of emotion of smile or laughter mistakenly co-fires the part of the III which dominants the levator palpebrae and made it contraction.Studies showed the expression of laughter seemed to depend on two partially independent neuronal pathways, the “involuntary” or “emotionally driven” system and the “voluntary” system.On closer inspection we noticed that in our patient, the upper eyelid of the right eye contracted only when the patient really wanted to smile or laugh and the contraction was not observed when the patient made fake smile expression or squinted hard to contract the orbicularis oculi muscle.

We conducted whole-exome sequencing (WES) of the patient and her parents to identify the genes known to cause ptosis. No phenotypic variation of the pathogenic single nucleotide variants and InDels was found, thus indicating that the observed ptosis was sporadic [15]. The most common cause of congenital ptosis is idiopathic; however, it can also be familial and transmitted in an autosomal dominant inheritance pattern. Some patients with congenital ptosis exhibit abnormal extraocular muscle innervation, known as congenital cranial dysinnervation disorders (CCDDs). Two genetic loci, namely PTOS1 and PTOS2, have been reported in patients with congenital blepharoptosis [16, 17]. In recent years, high-resolution MRI has been gradually used to diagnose CCDDs. The MRI scanning of the cisterna segment of the oculomotor nerve is now an established process. Clinical examinations combined with MRI and WES were performed to reveal the phenotypic and genotypic characteristics of CCDDs in some studies [18]. We also performed these tests on our patient and found no abnormity.

Although we wanted to use electromyography to identify abnormal facial synkinesis, the patient was 10 years of age and did not cooperate well, it was impossible for our patient to smile while tolerating the discomfort caused by electromyography. We also found that the conduction latency of the levator palpebrae in the right eye was longer than that in the left eye when the patient was calm; this finding indicated the LP of the right eye responded more slowly to stimulation than that of the left eye. A limitation is that there is currently no normal value for the levator palpebrae superioris muscle in electromyography for children, and we can draw conclusions only by comparing the results of both eyes.

Although congenital ptosis is a nonprogressive condition, surgery is required when patients show a tendency to have amblyopia. Ptosis treatment may involve resection of the involved muscle (levator muscle or Müller muscle) or suspension of the upper eyelid to the frontalis muscle. For ptosis with impaired innervation, surgery should aim to eliminate synkinesis and obtain a satisfactory and symmetrical eyelid opening. However, surgical methods have varied and remained controversial for several years. Our patient did not have amblyopia, and the impaired innervation showed a remission tendency, thus indicating this phenomenon of impaired innervation can gradually reduce with time without the requirement for surgical intervention. The myopia and astigmatism in her ptosis eye might be due to eyelid tension and changes in the corneal curvature [19].

## Conclusion

Here, we report a case of ptosis eye with impaired innervation. In this case, blepharoptosis was extremely insidious, and the contraction of the upper eyelid when smiling covered up right eyelid ptosis; the contractive right upper eyelid when smiling masked the weakness of the right levator muscle, thus creating a diagnostic challenge for clinicians, few of whom might consider this condition as right eye ptosis. For synkinesis observed in our case, impaired innervation should be considered. Levator muscle function and periocular MRI should be carefully examined to determine whether the condition is ptosis and whether it is secondary to periocular diseases to facilitate a clear diagnosis and subsequent treatment. Compared to the initial visit, the patient showed lesser eyelid retraction after 6 months of follow-up. We speculate that this type of impaired innervation with no systemic manifestations and mild symptoms can gradually reduce or even alleviate with time. We will continue our follow-up and further study on this topic.

### Electronic supplementary material

Below is the link to the electronic supplementary material.

Supplementary Material 1

## Data Availability

No datasets were generated or analysed during the current study.

## Abbreviations

WES: Whole-Exome Sequencing
MRI: Magnetic Resonance Imaging
RNS: Repetitive Nerve Stimulation
MGJW: Marcus Gunn jaw-winking Syndrome
CCDDs: Congenital Cranial Dysinnervation Disorders
