# Supplementary material for: Eyelid retraction during smiling in a patient with monocular congenital ptosis: a case report
Source: BMC Ophthalmol. 2024 May 31;24:232. doi: 10.1186/s12886-024-03485-8 (PMC11143675; doi:10.1186/s12886-024-03485-8)
Supplement: Supplementary file 1 — Supplementary Material 1 [file 12886_2024_3485_MOESM1_ESM.pdf]

Stim Side	Ipsi R1-Lat ms	Ipsi R2-Lat ms	Contra R2-Lat ms	Diff R2-Lat ms	Ipsi R1-Amp mV	Ipsi R2-Amp mV	Contra R2-Amp mV
Left	10.6	31.4	30.7	0.7	0.6	0.9	0.6
Right	10.1	30.5	29.9	0.6	0.4	0.3	0.7
	0.5	0.9	0.8		0.2	0.6	0.1

Group: 1 Stim: Left Rec: Ipsi Ch. 1
Group: 2 Stim: Left Rec: Contra Ch. 2
Group: 3 Stim: Right Rec: Ipsi Ch. 2
Group: 4 Stim: Right Rec: Contra Ch. 1

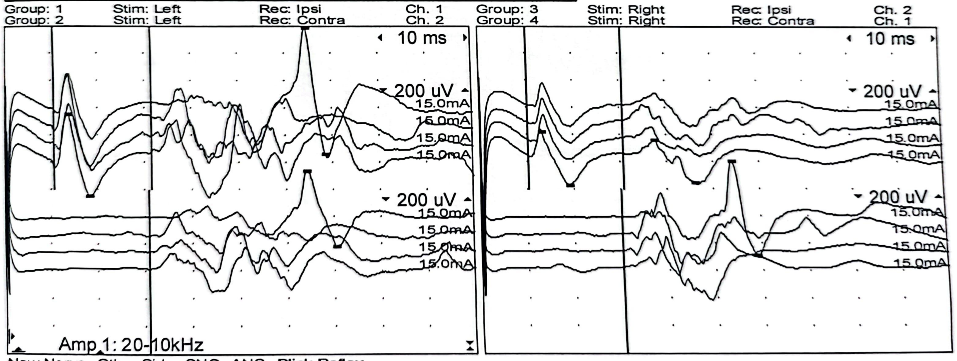

New Nerve Other Side SNC ANS **Blink Reflex**

Facial.L

提上睑肌	2.5 ms	1.1 mV	Orbicularis oculi-Anterior auricular	2.5 ms	95 mm	m/s

Facial.R

提上睑肌	2.8 ms	1.2 mV	Orbicularis oculi-Anterior auricular	2.8 ms	100 mm	m/s
